# Data of correlation analysis between the density of H3K4me3 in promoters of genes and gene expression: Data from RNA-seq and ChIP-seq analyses of the murine prefrontal cortex

**DOI:** 10.1016/j.dib.2020.106365

**Published:** 2020-10-02

**Authors:** V.V. Reshetnikov, P.E. Kisaretova, N.I. Ershov, T.I. Merkulova, N.P. Bondar

**Affiliations:** a Institute of Cytology and Genetics, Siberian Branch of Russian Academy of Sciences (SB RAS), Novosibirsk, Russia; b National Research Novosibirsk State University, Novosibirsk, Russia

**Keywords:** H3K4me3, RNA-seq, ChIP-seq, Prefrontal cortex, Epigenetics, Cell specificity

## Abstract

H3K4me3 is typically found in the promoter region of genes and is a mark associated with an open chromatin state and active gene transcription. Nonetheless, the role of H3K4me3 in the regulation of transcription is still debated. To improve the understanding of the connection between H3K4me3 density in promoters and gene expression, we assessed the correlation between these two parameters. We utilized genome-wide high-throughput RNA sequencing (RNA-seq) data and H3K4me3-based chromatin immunoprecipitation with high-throughput sequencing (ChIP-seq), carried out on the same samples of the prefrontal cortex from 10 male C57Bl6 mice with different stress experience [Social defeat stress in adult mice causes alterations in gene expression, alternative splicing, and the epigenetic landscape of H3K4me3 in the prefrontal cortex: an impact of early-life stress, 1]. In addition, we assessed the correlation between H3K4me3 density and gene expression in datasets of cell-specific genes. Altogether, the results are useful for the elucidation of H3K4me3 involvement in the regulation of transcription in the murine prefrontal cortex.

## Specifications Table

**Table:** 

Subject	Molecular Biology
Specific subject area	Epigenetics
Type of data	Tables, graphs
How data were acquired	RNA-seq and H3K4me3-based chromatin immunoprecipitation with sequencing (HiSeq 4000), bioinformatics
Data format	Raw
Parameters for data collection	Samples of the prefrontal cortex from 10 male C57Bl6 mice with different stress experience were used. Native micrococcal-nuclease-treated–chromatin immunoprecipitation with antibodies to H3K4me3 and total RNA extraction from the same homogenate of cortical tissue were performed.
Description of data collection	Paired-end (2 × 100 bp) sequencing for RNA-seq and single-end sequencing (1 × 100 bp) for ChIP-seq were performed on the Illumina HiSeq 4000 platform. On average, ∼ 31.8 million unpaired reads (18.4–41.4 million) were obtained for each sample of ChIP-seq library, and on average, ∼43 million paired-end reads (38–47 million) were obtained for each sample using Illumina stranded sequencing (RNA-seq). The RNA-seq dataset comprises 14,901 genes with at least 10 counts in each sample. In promoter regions (±1 kbp around a transcription start site) of these genes, 13,467 broad peaks and 73,454 single-nucleosome peaks of H3K4me3 were identified and used for correlation analysis.
Data source location	Institute of Cytology and Genetics, SB RAS, Prospekt Lavrentyeva 10, Novosibirsk, Russian Federation
Data accessibility	Raw data of ChIP-seq and RNA-seq were deposited in the NCBI BioProject database under the project ID PRJNA610193 (https://www.ncbi.nlm.nih.gov/bioproject/?term=PRJNA610193)
Related research article	Reshetnikov, V. V., Kisaretova, P. E., Ershov, N. I., Merkulova, T. I., & Bondar, N. P. (2020). Social defeat stress in adult mice causes alterations in gene expression, alternative splicing, and the epigenetic landscape of H3K4me3 in the prefrontal cortex: An impact of early-life stress. Progress in Neuro-Psychopharmacology and Biological Psychiatry, DOI:10.1016/j.pnpbp.2020.110068.

## Value of the Data

• Our data are useful for elucidation of the role of H3K4me3 marks in the regulation transcription in the murine prefrontal cortex
• The reported dataset is the combined data on murine prefrontal cortex RNA-seq and H3K4me3 native-ChIP-seq performed on the same individuals, which allows to trace the relations between characteristics of an H3K4me3 signal and mRNA expression at the level of individual genes.
• Correlation analysis of H3K4me3 density and gene expression elucidates the regulation of transcription of cell-specific genes.
• The resolution of native ChIP-seq analysis enables to examine the impact of H3K4me3 amounts in individual nucleosomes on expression efficiency of the corresponding gene in the prefrontal cortex.

## 1 Data Description

**Fig. 1:**
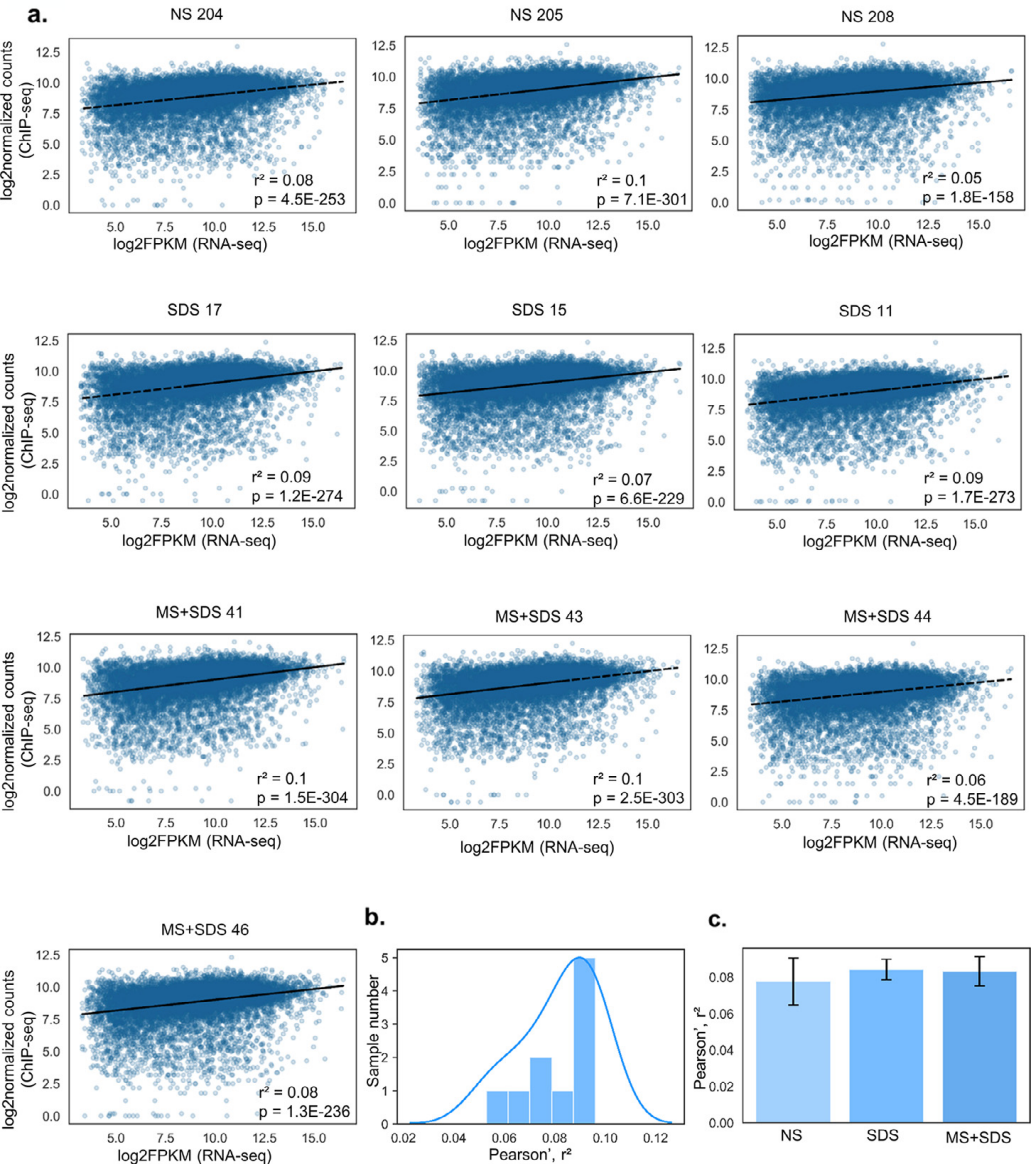
Correlation between density of broad H3K4me3 peaks in promoters and gene expression for each sample.

**Fig. 2:**
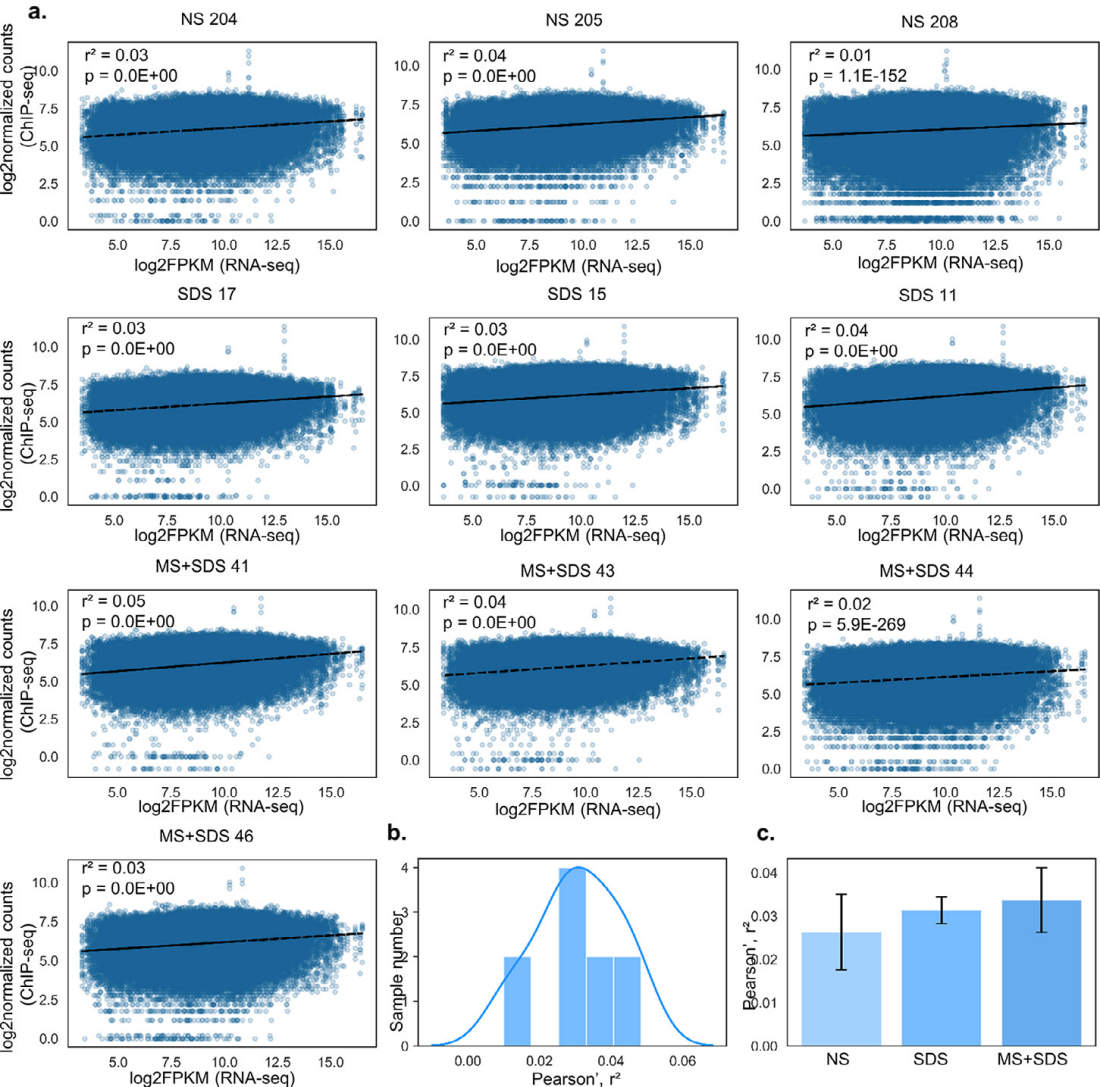
Correlation between density of single-nucleosome H3K4me3 peaks in promoters and gene expression for each sample.

Analysis of the correlation between broad H3K4me3 peaks in promoters of genes detected by RNA-seq here and gene expression in 10 individual samples revealed only low correlation between these two parameters: r^2^ = 0.082 (0.052–0.098, Fig. 1). Similarly to broad peaks, analysis of correlation between single-nucleosome peaks of H3K4me3 and gene expression also showed low correlation: r^2^ = 0.031 (0.052–0.098, Fig. 2). Correlation coefficients for both broad and single-nucleosome H3K4me3 peaks computed separately for each sample were normally distributed and did not show dependence on previous stressful experience [one-way ANOVA, broad peaks: F(2, 9) = 0.19, *p* = 0.83; single-nucleosome peaks: F(2,9) = 0.43, *p* = 0.66].

**a.** The scatter plots illustrate relations between log_2_-normalized counts of H3K4me3 distribution in promoter regions (ChIP-seq) and gene expression (log_2_FPKM data from RNA-seq analysis) for each sample. **b.** Distribution of Pearson's r² values among the samples. **c.** Pearson's r² means by experimental group. Data are presented as mean ± SEM. NS: mice with normal rearing early in life and unstressed in adulthood, SDS: mice with normal rearing early in life and SDS in adulthood, MS+SDS: mice with a history of maternal separation early in life and SDS in adulthood.

**a.** The scatter plots illustrate relations between log_2_-normalized counts of H3K4me3 distribution in promoter regions (ChIP-seq) and gene expression (log_2_FPKM data from RNA-seq analysis) for each sample. **b.** Distribution of Pearson's r² values among the samples. **c.** Pearson's r² means by experimental group. Data are presented as mean ± SEM. NS: mice with normal rearing early in life and unstressed in adulthood, SDS: mice with normal rearing early in life and SDS in adulthood, MS+SDS: mice with a history of maternal separation early in life and SDS in adulthood.

We found that only 5.1% (688) of broad peaks in promoters correlate with gene expression (*p* < 0.05), and 372 of them manifested negative whereas 316 positive correlation (Supplement T1). Similarly to broad peaks of H3K4me3, only 4.8% (3528) single-nucleosome peaks correlate significantly (*p* < 0.05), with 54% (1904) of them showing negative correlation, and 46% (1624) positive (Supplement T1). Multiple-comparison p-value correction by the Benjamini–Hochberg method did not leave any significant correlations.

**Fig. 3:**
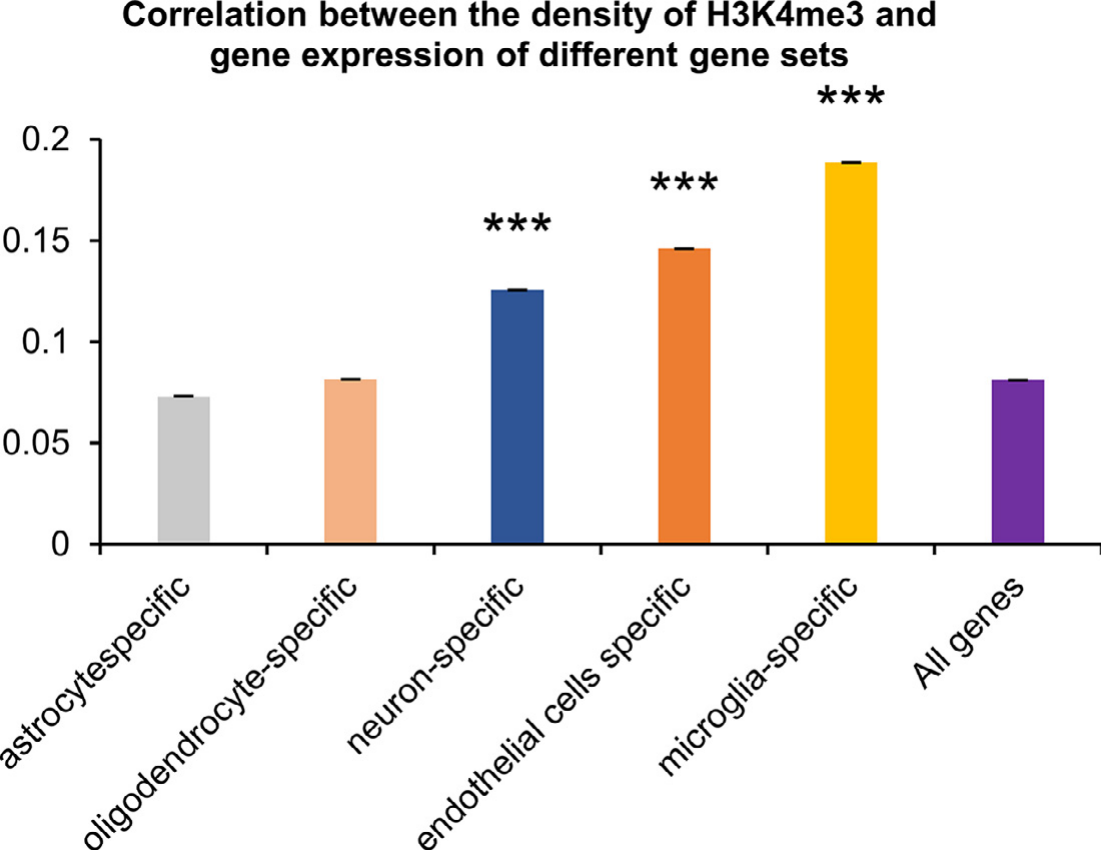
Correlation between density of broad H3K4me3 peaks and gene expression in the datasets of cell-specific genes.

Next, the correlation of H3K4me3 peaks among cell-specific genes that are expressed predominantly in astrocytes, endothelial cells, microglia, neurons, or oligodendrocytes was analyzed. We found a significant dependence of this correlation on the cell type [one-way ANOVA: F(5, 55) = 41.43, *p* < 0.001, Fig. 3, Supplement T2]. Coefficients of correlation (r^2^) between broad H3K4me3 peaks in promoters of cell-specific genes did not depend on the group of animals and varied between 0.073 and 0.189. H3K4me3 density correlated more strongly with gene expression among microglia-specific genes compared to other cell-specific genes (*p* < 0.05). Expression of neuron- and endothelial-cell–specific genes also correlates more strongly with H3K4me3 density as compared to the correlation coincident of all genes detected by RNA-req here (r^2^ = 0.126 and r^2^ = 0.146, *p* < 0.001).

Correlation coefficients in different datasets of cell-specific genes. Data are presented as mean ± SEM. ***p < 0.001 as compared with all genes under study.

## 2 Experimental Design

### 2.1 ChIP, total-RNA extraction and library construction

The samples of prefrontal cortex from 10 male C57Bl6 mice with a different history of stress were used for ChIP-seq and RNA-seq analysis [1]*.* Briefly, each sample of the prefrontal cortex was homogenized and one aliquot of the homogenate was used for total RNA extraction, the rest of the homogenate was used for ChIP as described previously [1]. ChIP with an antibody to H3K4me3 (ab8580, Abcam) was carried out according to a previously described method [2] with some modifications [3]. Micrococcal nuclease digestion (NEB cat. # M0247S) was used. For the RNA-seq analysis, only samples with the RNA integrity number (RIN) > 8.0 were used.

ChIP-seq and RNA-seq libraries were prepared in accordance with New England Biolab protocols (NEB, USA) as previously described [1]. The size and quantity of each library were verified on an Agilent Bioanalyzer. Paired-end (2 × 100 bp) sequencing for RNA-seq and single-end sequencing (1 × 100 bp) for ChIP-seq were performed on the Illumina HiSeq 4000 platform (Evrogen Joint Stock Company, Russia). Raw data were deposited in the NCBI BioProject database under the project ID PRJNA610193 (https://www.ncbi.nlm.nih.gov/bioproject/?term=PRJNA610193).

### 2.2 RNA-seq data analysis

Ten stranded RNA-seq libraries were sequenced on the Illumina HiSeq 4000 platform to a depth of 38–47 million 100 bp paired-end reads. Low-quality and adapter sequences were removed from the FASTQ-formatted data with the Trimmomatic 0.36 tool [4]. The surviving sequences were mapped to the *Mus musculus* GRCm38/mm10 reference genome assembly by means of the splice-aware HISAT2 aligner (version 2.1.0) [5]. Bases mapped to transcripts (i.e., coding and untranslated regions) constituted 86.4–92.5% of all bases, and only 0.4% of bases were found to map to ribosomal sequences (0.2–0.8%, for a detailed explanation, see Supplement materials [1]). The mapped data were summarized as gene level log_2_(FPKM) values using the GENCODE gene annotation (release M13) and a custom R script via the RLE (relative log expression) procedure from the DESeq2 [6] R package.

### 2.3 ChIP-seq data analysis

Ten N—ChIP-seq libraries were sequenced using the Illumina HiSeq 4000 platform to a depth of 18.4–41.4 million 100 bp single-end reads. The data were similarly processed by the Trimmomatic tool and mapped to the GRCm38/mm10 reference genome in the bowtie2 aligner [7]. On average, 67.2% (62.7–74.1%) reads turned out to be mapped uniquely. All the libraries except two (with Qtag = 1) got a Qtag score of 2 in a quality check by the Phantompeakqualtools software [8]. The MACS2 software [9] was employed to call peaks in the aligned data versus the input library in two layouts: (i) broad beaks of varied length (–broad switch) and (ii) single-nucleosome positions (–call-summits switch), which were subsequently extended up to 147 bp intervals. The rest of the MACS2 parameters were “-g mm –keep-dup all –nomodel –shift 37 –extsize 73.” For each library, read counts computed for broad and single-nucleosome peaks were normalized to the sequencing depth via the RLE procedure from DESeq2 and were log_2_-transformed. We further analyzed only H3K4me3 peaks located in promoter regions of the genes (±1 kbp around a transcription start site) that were detected by RNA-seq in this study.

### 2.4 Correlation analysis

The use of micrococcal nuclease in the chromatin immunoprecipitation together with high depth of sequencing allowed us to identify both broad peaks and nucleosome-sized peaks. We assessed the correlation between density of broad and single-nucleosome H3K4me3 peaks in promotors and gene expression using Pearson's correlation coefficient. Normalized expression values in FPKM (reads per kilobase of exon per million fragments mapped) of 14,901 genes with at least 10 counts in each sample and density of ChIP-seq reads (normalized counts) in promoters (±1 kbp around a transcription start site) were used (Supplement material 3). In addition, we evaluated correlation between broad H3K4me3 peaks in promoters of cell-specific genes [10].

### 2.5 Statistical analysis

Normality of data distribution and homogeneity of variances were tested via the Shapiro–Wilk and Levene tests, respectively. Because most of the data were normally distributed, one-way ANOVA was conducted, and Bonferroni's least significant difference (LSD) test was performed as *post hoc* analysis. The STATISTICA 8 software was employed for all the statistical analyses. A p value less than 0.05 was assumed to indicate significance.

## Ethical Statement

C57BL/6 mice were maintained at the Animal Facility of the Institute of Cytology and Genetics, SB RAS, Novosibirsk, Russia (RFMEFI62117 × 0015). All procedures were approved by the Ethical Committee of the Institute of Cytology and Genetics, SB RAS (Protocol #25, December 2014).

## Declaration of Competing Interest

None.

## Appendix Supplementary materials

Image, application 1

Image, application 2

Image, application 3
