# Supplementary material for: Data of correlation analysis between the density of H3K4me3 in promoters of genes and gene expression: Data from RNA-seq and ChIP-seq analyses of the murine prefrontal cortex
Source: Data Brief. 2020 Oct 2;33:106365. doi: 10.1016/j.dib.2020.106365 (PMC7575808; doi:10.1016/j.dib.2020.106365)
Supplement: Supplementary file 3 [file mmc3.pdf]

In [1]:
```
import pandas as pd
import numpy as np
import matplotlib.pyplot as plt
import scipy.stats
import statsmodels.stats.multitest
```

In [2]:
```
rna = pd.read_table('ex14_filt10counts_R_SS_PFC_CTSS_vs_CTCT.txt')
chip = pd.read_table('diffpeaks_prom_ex14_R_PKB_SS_CT.Q10.BLmasked.txt'
)
chip_s = pd.read_table('diffpeaks_prom_ex14_R_PKS_SS_CT.Q10.BLmasked.tx
t')
```

In [3]:
```
chip.count()[0]
```

Out[3]:
```
14660
```

In [4]:
```
#genes in RNAseq
needed = list(rna['gene_name'])
#RNAseq
rna_by_gene = rna[['gene_name',
 'K_RNA_204',
 'K_RNA_205',
 'K_RNA_208',
 'K_SS_RNA_11',
 'K_SS_RNA_15',
 'K_SS_RNA_17',
 'MS_SS_RNA_41',
 'MS_SS_RNA_43',
 'MS_SS_RNA_44',
 'MS_SS_RNA_46']]
```

In [5]:
```
len(set(needed))
```

Out[5]:
```
14896
```

Create PDF in your applications with the Pdfcrowd HTML to PDF API PDFCROWD

In [6]:
```
len(needed)
```

Out[6]:
```
14901
```

In [7]:
```
#correlation of RNAseq vs ChIPseq by gene NUCLEOSOMAL peaks data
chip_s_by_gene = chip_s[['Unnamed: 0', 'gene_locs','gene_names',
 'K_RNA_204',
 'K_RNA_205',
 'K_RNA_208',
 'K_SS_RNA_11',
 'K_SS_RNA_15',
 'K_SS_RNA_17',
 'MS_SS_RNA_41',
 'MS_SS_RNA_43',
 'MS_SS_RNA_44',
 'MS_SS_RNA_46']]
print('peaks '+str(chip_s_by_gene.count()[0]))

a0=[]
a1=[]
a2=[]
a3=[]
a4=[]
a5=[]
a6=[]
a7=[]
a8=[]
a9=[]
a10=[]
a11=[]
a12=[]
for i, row in chip_s_by_gene.iterrows():
    for g in row['gene_names'].split(';'):
        if g in needed:
            a0.append(row['Unnamed: 0'])
            a1.append(row['gene_locs'])
            a2.append(g)
            a3.append(row['K_RNA_204'])
            a4.append(row['K_RNA_205'])
```

Create PDF in your applications with the Pdfcrowd HTML to PDF API PDFCROWD

```
            a5.append(row['K_RNA_208'])
            a6.append(row['K_SS_RNA_11'])
            a7.append(row['K_SS_RNA_15'])
            a8.append(row['K_SS_RNA_17'])
            a9.append(row['MS_SS_RNA_41'])
            a10.append(row['MS_SS_RNA_43'])
            a11.append(row['MS_SS_RNA_44'])
            a12.append(row['MS_SS_RNA_46'])
chip_s_by_gene1 = {'Unnamed: 0':a0, 'gene_locs':a1,'gene_name':a2,
 'K_RNA_204_ch':a3,
 'K_RNA_205_ch':a4,
 'K_RNA_208_ch':a5,
 'K_SS_RNA_11_ch':a6,
 'K_SS_RNA_15_ch':a7,
 'K_SS_RNA_17_ch':a8,
 'MS_SS_RNA_41_ch':a9,
 'MS_SS_RNA_43_ch':a10,
 'MS_SS_RNA_44_ch':a11,
 'MS_SS_RNA_46_ch':a12}
chip_s_by_gene1 = pd.DataFrame(chip_s_by_gene1)

print('peaks extended by gene'+str(chip_s_by_gene1.count()[0])+' genes
 '+str(len(set(chip_s_by_gene1['gene_name']))))


#LEAVE GENES FROM RNASEQ (and ADD TO RNA DF DATA FOR CHIPSEQ LOG2BASEME
AN) BROAD BY GENE
by_gene_rna_nucl = pd.merge(rna_by_gene, chip_s_by_gene1, right_on = 'g
ene_name', left_on = 'gene_name')

print('peaks in RNAseq '+str(by_gene_rna_nucl.count()[0])+' genes vs RN
Aseq '+str(len(set(by_gene_rna_nucl['gene_name']))))

#CORRELATION BY GENE NUCLEOSOMAL
coln1 = [ 'K_RNA_204',
 'K_RNA_205',
 'K_RNA_208',
 'K_SS_RNA_11',
 'K_SS_RNA_15',
```

Create PDF in your applications with the Pdfcrowd HTML to PDF API PDFCROWD

```
 'K_SS_RNA_17',
 'MS_SS_RNA_41',
 'MS_SS_RNA_43',
 'MS_SS_RNA_44',
 'MS_SS_RNA_46']
coln2 = [ 'K_RNA_204_ch',
 'K_RNA_205_ch',
 'K_RNA_208_ch',
 'K_SS_RNA_11_ch',
 'K_SS_RNA_15_ch',
 'K_SS_RNA_17_ch',
 'MS_SS_RNA_41_ch',
 'MS_SS_RNA_43_ch',
 'MS_SS_RNA_44_ch',
 'MS_SS_RNA_46_ch']

cor = []
pval = []
for i, row in by_gene_rna_nucl.iterrows():
    x = [row[k] for k in coln1]
    y = [row[k] for k in coln2]
    st = scipy.stats.pearsonr(x,y)
    cor.append(st[0])
    pval.append(st[1])
by_gene_rna_nucl['Pearson_cor_R'] = cor
by_gene_rna_nucl['Pearson_cor_pval'] = pval
by_gene_rna_nucl['Pearson_cor_padj'] = statsmodels.stats.multitest.mult
ipletests(list(by_gene_rna_nucl['Pearson_cor_pval']), method='fdr_bh')[
1]
```

```
peaks 87489
peaks extended by gene73433 genes 12917
peaks in RNAseq 73454 genes vs RNAseq 12917
```

In [8]:
```
by_gene_rna_nucl
```

Out[8]:

	gene_name	K_RNA_204	K_RNA_205	K_RNA_208	K_SS_RNA_11	K_SS_RNA_15	K_SS_
0	Izumo4	280.443370	325.570300	302.694497	454.546689	514.327388	44

Create PDF in your applications with the Pdfcrowd HTML to PDF API PDFCROWD

	gene_name	K_RNA_204	K_RNA_205	K_RNA_208	K_SS_RNA_11	K_SS_RNA_15	K_SS_
1	Izumo4	280.443370	325.570300	302.694497	454.546689	514.327388	44
2	Izumo4	280.443370	325.570300	302.694497	454.546689	514.327388	44
3	Izumo4	280.443370	325.570300	302.694497	454.546689	514.327388	44
4	Izumo4	280.443370	325.570300	302.694497	454.546689	514.327388	44
...	...	...	...	...	...	...	
73449	Cdk16	6035.431746	4944.219767	5078.671870	5543.202023	5114.700132	490
73450	Cdk16	6035.431746	4944.219767	5078.671870	5543.202023	5114.700132	490
73451	Zranb1	1718.963568	1926.122424	1990.481322	1723.360689	2093.883942	188
73452	Zranb1	1718.963568	1926.122424	1990.481322	1723.360689	2093.883942	188
73453	Zranb1	1718.963568	1926.122424	1990.481322	1723.360689	2093.883942	188

73454 rows × 26 columns

In [9]:

```
#correlation of RNAseq vs ChIPseq by gene broad peaks data
chip_by_gene = chip[['Unnamed: 0', 'gene_locs','gene_names',
 'K_RNA_204',
 'K_RNA_205',
 'K_RNA_208',
 'K_SS_RNA_11',
 'K_SS_RNA_15',
 'K_SS_RNA_17',
 'MS_SS_RNA_41',
 'MS_SS_RNA_43',
 'MS_SS_RNA_44',
 'MS_SS_RNA_46']]
print('peaks '+str(chip_by_gene.count()[0]))
a0=[]
a1=[]
a2=[]
a3=[]
a4=[]
```

Create PDF in your applications with the Pdfcrowd HTML to PDF API PDFCROWD

```
a5=[]
a6=[]
a7=[]
a8=[]
a9=[]
a10=[]
a11=[]
a12=[]
for i, row in chip_by_gene.iterrows():
    for g in row['gene_names'].split(';'):
        if g in needed:
            a0.append(row['Unnamed: 0'])
            a1.append(row['gene_locs'])
            a2.append(g)
            a3.append(row['K_RNA_204'])
            a4.append(row['K_RNA_205'])
            a5.append(row['K_RNA_208'])
            a6.append(row['K_SS_RNA_11'])
            a7.append(row['K_SS_RNA_15'])
            a8.append(row['K_SS_RNA_17'])
            a9.append(row['MS_SS_RNA_41'])
            a10.append(row['MS_SS_RNA_43'])
            a11.append(row['MS_SS_RNA_44'])
            a12.append(row['MS_SS_RNA_46'])
chip_by_gene1 = {'Unnamed: 0':a0, 'gene_locs':a1,'gene_name':a2,
 'K_RNA_204_ch':a3,
 'K_RNA_205_ch':a4,
 'K_RNA_208_ch':a5,
 'K_SS_RNA_11_ch':a6,
 'K_SS_RNA_15_ch':a7,
 'K_SS_RNA_17_ch':a8,
 'MS_SS_RNA_41_ch':a9,
 'MS_SS_RNA_43_ch':a10,
 'MS_SS_RNA_44_ch':a11,
 'MS_SS_RNA_46_ch':a12}
chip_by_gene1 = pd.DataFrame(chip_by_gene1)

print('peaks extended by gene in RNAseq'+str(chip_by_gene1.count()[0])+
' genes '+str(len(set(chip_by_gene1['gene_name']))))
```

Create PDF in your applications with the Pdfcrowd HTML to PDF API PDFCROWD

```
#LEAVE GENES FROM RNASEQ (and ADD TO RNA DF DATA FOR CHIPSEQ LOG2BASEME
AN) BROAD BY GENE
by_gene_rna_broad = pd.merge(rna_by_gene, chip_by_gene1, right_on = 'ge
ne_name', left_on = 'gene_name')

print('peaks in RNAseq '+str(by_gene_rna_broad.count()[0])+' genes vs R
NAseq '+str(len(set(by_gene_rna_broad['gene_name']))))

#CORRELATION BY GENE
coln1 = [ 'K_RNA_204',
 'K_RNA_205',
 'K_RNA_208',
 'K_SS_RNA_11',
 'K_SS_RNA_15',
 'K_SS_RNA_17',
 'MS_SS_RNA_41',
 'MS_SS_RNA_43',
 'MS_SS_RNA_44',
 'MS_SS_RNA_46']
coln2 = [ 'K_RNA_204_ch',
 'K_RNA_205_ch',
 'K_RNA_208_ch',
 'K_SS_RNA_11_ch',
 'K_SS_RNA_15_ch',
 'K_SS_RNA_17_ch',
 'MS_SS_RNA_41_ch',
 'MS_SS_RNA_43_ch',
 'MS_SS_RNA_44_ch',
 'MS_SS_RNA_46_ch']

cor = []
pval = []
for i, row in by_gene_rna_broad.iterrows():
    x = [row[k] for k in coln1]
    y = [row[k] for k in coln2]
    st = scipy.stats.pearsonr(x,y)
    cor.append(st[0])
    pval.append(st[1])
```

Create PDF in your applications with the Pdfcrowd HTML to PDF API PDFCROWD

```
by_gene_rna_broad['Pearson_cor_R'] = cor
by_gene_rna_broad['Pearson_cor_pval'] = pval
by_gene_rna_broad['Pearson_cor_padj'] = statsmodels.stats.multitest.mul
tipletests(list(by_gene_rna_broad['Pearson_cor_pval']), method='fdr_bh'
)[1]
```

```
peaks 14660
peaks extended by gene in RNAseq13463 genes 12930
peaks in RNAseq 13467 genes vs RNAseq 12930
```

In [81]:
```
from decimal import Decimal
```

In [103]:
```
def cor_log(a, b, name):

    x = np.log2(a)
    #.replace([np.inf, -np.inf], 0)
    y = np.log2(b).replace([np.inf, -np.inf], 0)
    print(name)
    print(f'r\N{SUPERSCRIPT TWO} = '+str(round(np.power(scipy.stats.pea
rsonr(x,y)[0],2), 2))+'\n'+'p = '+str('%.1E' % Decimal(scipy.stats.pear
sonr(x,y)[1])))

    #GRAPH
    plt.figure(figsize = [4, 2.5])
    plt.scatter(x, y, c = '#176396', alpha = 0.2, s = 10)
    # calc the trendline
    z = np.polyfit(x, y, 1)
    p = np.poly1d(z)
    plt.plot(x,p(x),"k--")
    # the line equation:
    print("y=%.6fx+(%.6f)"%(z[0],z[1]))

    #plt.title(name)
    plt.savefig(f'{name}.png', transparent=True, bbox_inches="tight", d
pi = 500)
```

In [104]:
```
#broad graph
coln1 = [ 'K_RNA_204',
```

Create PDF in your applications with the Pdfcrowd HTML to PDF API PDFCROWD

```
 'K_RNA_205',
 'K_RNA_208',
 'K_SS_RNA_11',
 'K_SS_RNA_15',
 'K_SS_RNA_17',
 'MS_SS_RNA_41',
 'MS_SS_RNA_43',
 'MS_SS_RNA_44',
 'MS_SS_RNA_46']
coln2 = [ 'K_RNA_204_ch',
 'K_RNA_205_ch',
 'K_RNA_208_ch',
 'K_SS_RNA_11_ch',
 'K_SS_RNA_15_ch',
 'K_SS_RNA_17_ch',
 'MS_SS_RNA_41_ch',
 'MS_SS_RNA_43_ch',
 'MS_SS_RNA_44_ch',
 'MS_SS_RNA_46_ch']

for i in range(0, len(coln1)):
    cor_log(by_gene_rna_broad[coln1[i]],by_gene_rna_broad[coln2[i]], 'B
road_'+str(coln1[i]))
```

```
Broad_K_RNA_204
r² = 0.08
p = 4.5E-253
y=0.167745x+(7.322526)
Broad_K_RNA_205
r² = 0.1
p = 7.1E-301
y=0.176738x+(7.271793)
Broad_K_RNA_208
r² = 0.05
p = 1.8E-158
y=0.140068x+(7.542810)
Broad_K_SS_RNA_11
r² = 0.09
p = 1.7E-273
y=0.176430x+(7.259124)
```

Create PDF in your applications with the Pdfcrowd HTML to PDF API PDFCROWD

```
Broad_K_SS_RNA_15
r² = 0.07
p = 6.6E-229
y=0.170867x+(7.284707)
Broad_K_SS_RNA_17
r² = 0.09
p = 1.2E-274
y=0.191377x+(7.101344)
Broad_MS_SS_RNA_41
r² = 0.1
p = 1.5E-304
y=0.199655x+(7.032196)
Broad_MS_SS_RNA_43
r² = 0.1
p = 2.5E-303
y=0.185049x+(7.202430)
Broad_MS_SS_RNA_44
r² = 0.06
p = 4.5E-189
y=0.157556x+(7.389004)
Broad_MS_SS_RNA_46
r² = 0.08
p = 1.3E-236
y=0.166599x+(7.335256)
```

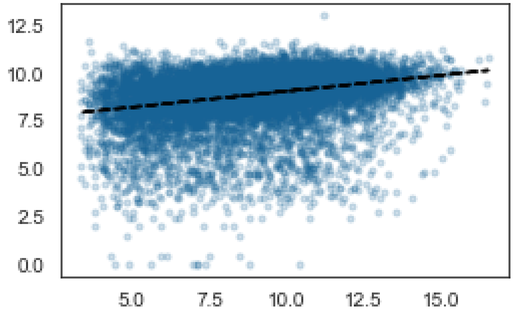

Create PDF in your applications with the Pdfcrowd HTML to PDF API PDFCROWD

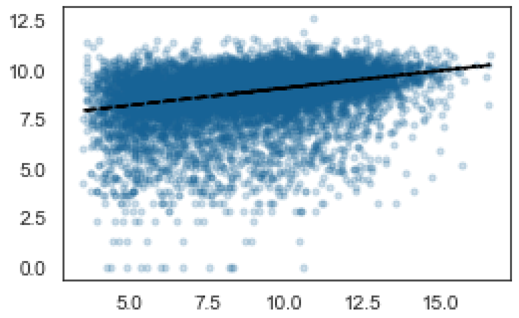

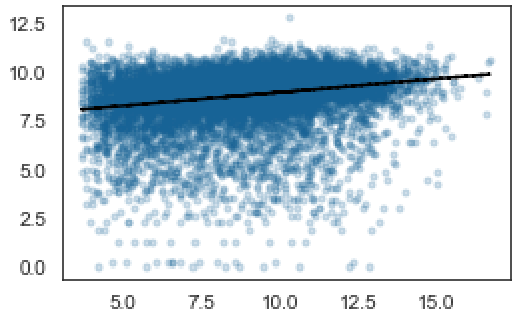

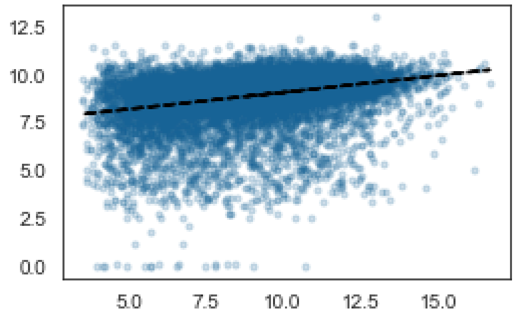

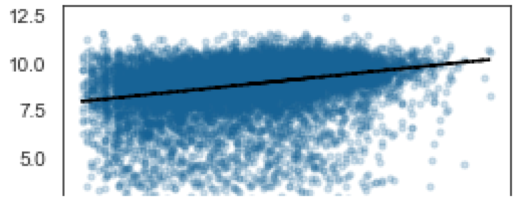

Create PDF in your applications with the Pdfcrowd HTML to PDF API PDFCROWD

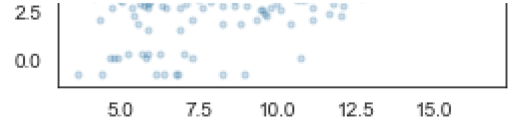

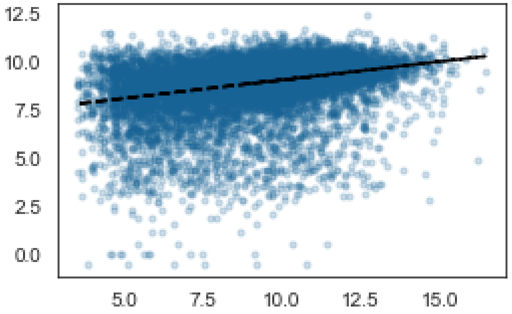

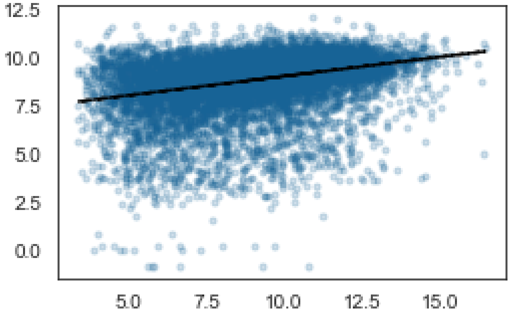

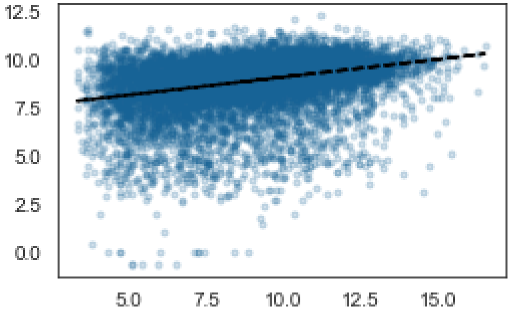

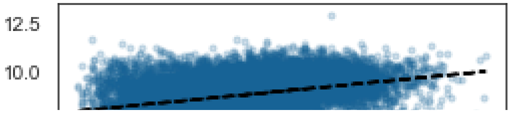

Create PDF in your applications with the Pdfcrowd HTML to PDF API PDFCROWD

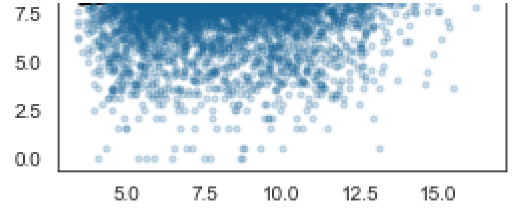

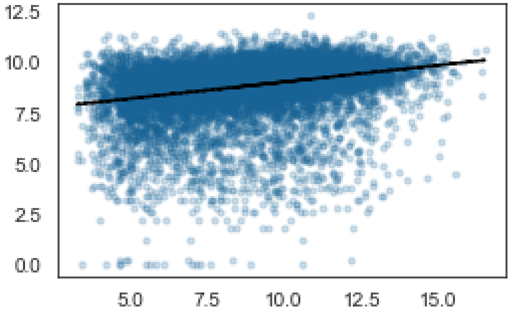

In [98]:
```
broad_r = pd.Series(np.power([0.29,0.31,0.23,0.3,0.27,0.3,0.31,0.31,0.2
5,0.28], 2))
nucl_r = pd.Series(np.power([0.17,0.2,0.1,0.18,0.16,0.19,0.22,0.21,0.13
,0.16],2))
```

In [99]:
```
plt.figure(figsize = [4, 2.5])


names = ['NS', 'SDS', 'MS+SDS']
color = [(101,179,255), '#1e90ff', '#0074e4']
plt.bar('NS', broad_r[0:3].mean(), color = '#65b3ff', alpha = 0.6)
plt.errorbar(0, broad_r[0:3].mean(), broad_r[0:3].sem(), color = 'blac
k', capsize=4)
plt.bar('SDS', broad_r[3:6].mean(), color = '#1e90ff', alpha = 0.6)
plt.errorbar(1, broad_r[3:6].mean(), broad_r[3:6].sem(), color = 'blac
```

Create PDF in your applications with the Pdfcrowd HTML to PDF API PDFCROWD

```
k', capsize=4)
plt.bar('MS+SDS', broad_r[6:10].mean(), color = '#0074e4', alpha = 0.6)
plt.errorbar(2, broad_r[6:10].mean(), broad_r[6:10].sem(), color = 'bla
ck', capsize=4)

plt.savefig('mean_r_broad.png', transparent=True, bbox_inches="tight",
dpi = 500)
```

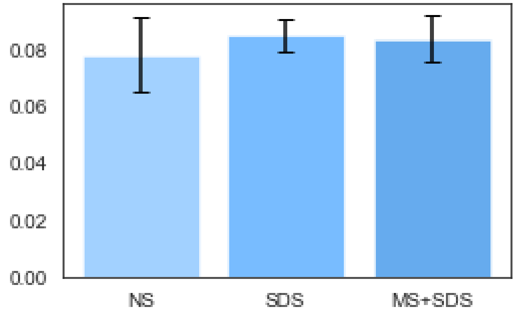

In [100]:
```
plt.figure(figsize = [4, 2.5])


names = ['NS', 'SDS', 'MS+SDS']
color = [(101,179,255), '#1e90ff', '#0074e4']
plt.bar('NS', nucl_r[0:3].mean(), color = '#65b3ff', alpha = 0.6)
plt.errorbar(0, nucl_r[0:3].mean(), nucl_r[0:3].sem(), color = 'black',
 capsize=4)
plt.bar('SDS', nucl_r[3:6].mean(), color = '#1e90ff', alpha = 0.6)
plt.errorbar(1, nucl_r[3:6].mean(), nucl_r[3:6].sem(), color = 'black',
 capsize=4)
plt.bar('MS+SDS', nucl_r[6:10].mean(), color = '#0074e4', alpha = 0.6)
plt.errorbar(2, nucl_r[6:10].mean(), nucl_r[6:10].sem(), color = 'blac
k', capsize=4)

plt.savefig('mean_r_nucl.png', transparent=True, bbox_inches="tight", d
pi = 500)
```

Create PDF in your applications with the Pdfcrowd HTML to PDF API PDFCROWD

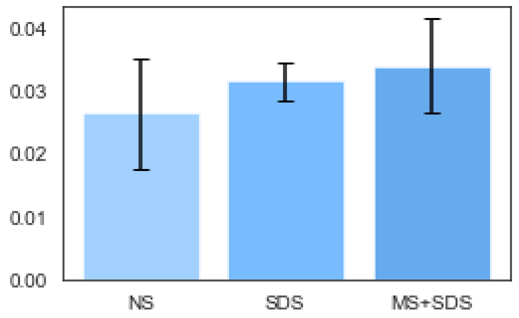

In [21]:
```
plt.figure(figsize=(10,6))
x = pd.Series(broad_r)
mu = x.mean() # mean of distribution
sigma = x.std() # standard deviation of distribution
num_bins = 5
# the histogram of the data
n, bins, patches = plt.hist(x, num_bins, density=True, facecolor='blue'
, alpha=0.5)

# add a 'best fit' line
y = mlab.normpdf(bins, mu, sigma)
plt.plot(bins, y, 'r--')
plt.xlabel('Pearson, R')

# Tweak spacing to prevent clipping of ylabel
plt.subplots_adjust(left=0.15)

plt.yticks([])
plt.savefig('broad_cor_by_gene.png', transparent=True, bbox_inches="tig
ht")
```

```
C:\ProgramData\Anaconda3\lib\site-packages\ipykernel_launcher.py:11: Ma
tplotlibDeprecationWarning: scipy.stats.norm.pdf
  # This is added back by InteractiveShellApp.init_path()
```

Create PDF in your applications with the Pdfcrowd HTML to PDF API PDFCROWD

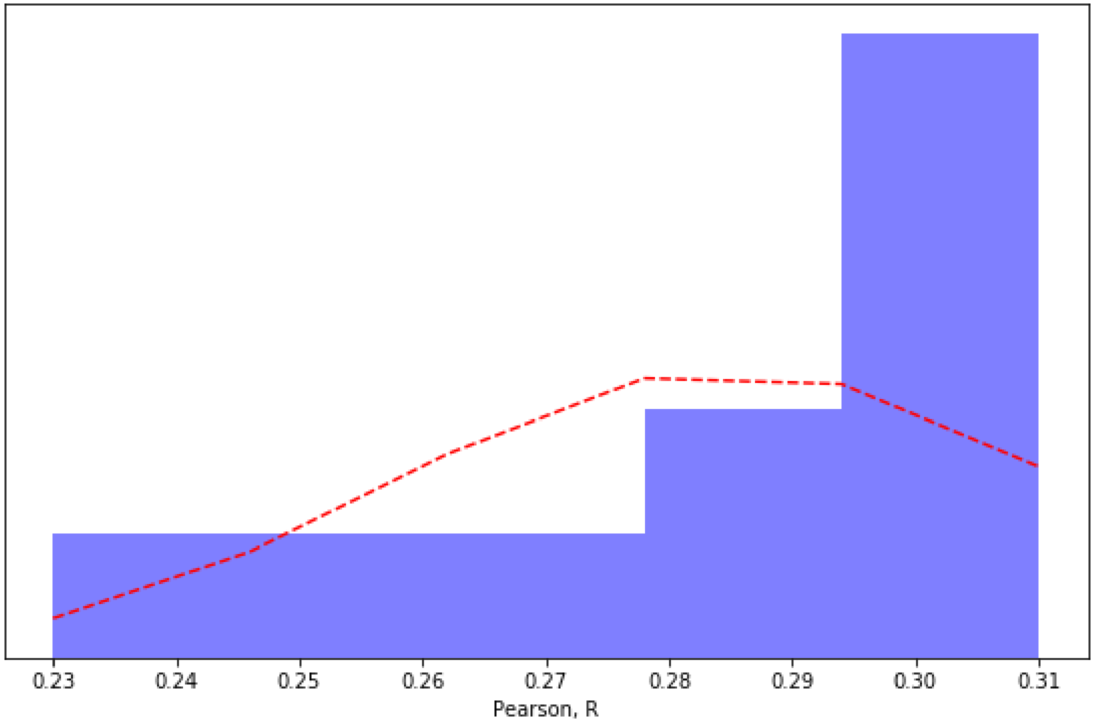

In [105]:
```
#Nucleosomal graphs
for i in range(0, len(coln1)):
    cor_log(by_gene_rna_nucl[coln1[i]],by_gene_rna_nucl[coln2[i]], 'Nuc
leosomal_'+str(coln1[i]))
```

```
Nucleosomal_K_RNA_204
r² = 0.03
p = 0.0E+00
y=0.088985x+(5.281705)
Nucleosomal_K_RNA_205
r² = 0.04
p = 0.0E+00
y=0.088190x+(5.351030)
Nucleosomal_K_RNA_208
```

Create PDF in your applications with the Pdfcrowd HTML to PDF API PDFCROWD

```
r² = 0.01
p = 1.1E-152
y=0.064779x+(5.378870)
Nucleosomal_K_SS_RNA_11
r² = 0.03
p = 0.0E+00
y=0.090958x+(5.305796)
Nucleosomal_K_SS_RNA_15
r² = 0.03
p = 0.0E+00
y=0.092201x+(5.256075)
Nucleosomal_K_SS_RNA_17
r² = 0.04
p = 0.0E+00
y=0.112492x+(5.062305)
Nucleosomal_MS_SS_RNA_41
r² = 0.05
p = 0.0E+00
y=0.115325x+(5.085879)
Nucleosomal_MS_SS_RNA_43
r² = 0.04
p = 0.0E+00
y=0.096842x+(5.300376)
Nucleosomal_MS_SS_RNA_44
r² = 0.02
p = 5.9E-269
y=0.077346x+(5.364109)
Nucleosomal_MS_SS_RNA_46
r² = 0.03
p = 0.0E+00
y=0.085964x+(5.311313)
```

Create PDF in your applications with the Pdfcrowd HTML to PDF API PDFCROWD

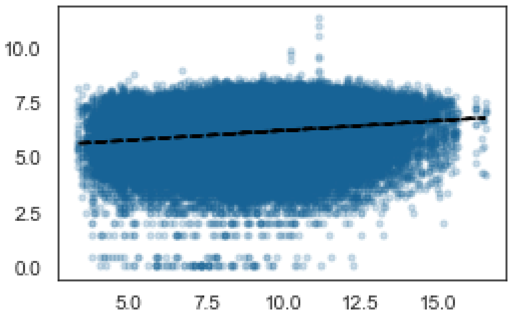

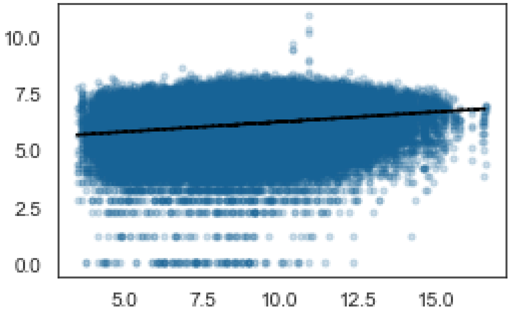

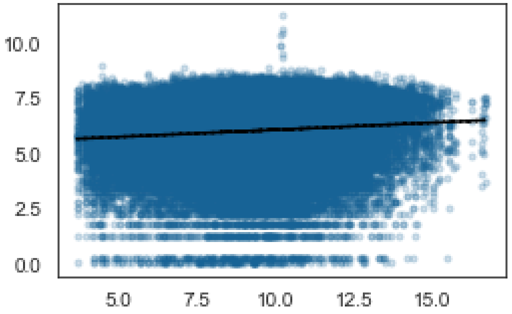

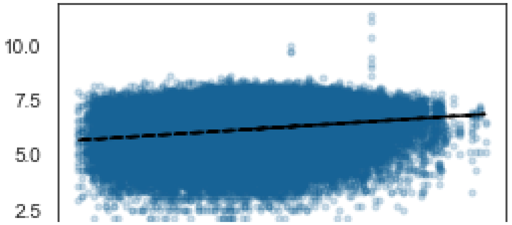

Create PDF in your applications with the Pdfcrowd HTML to PDF API PDFCROWD

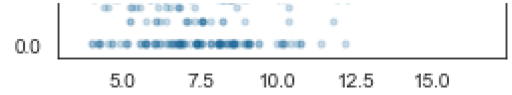

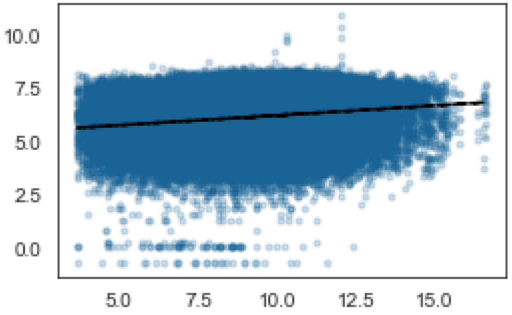

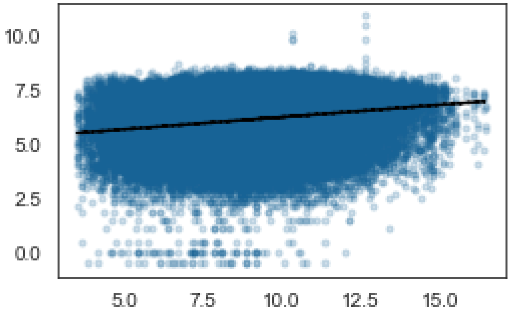

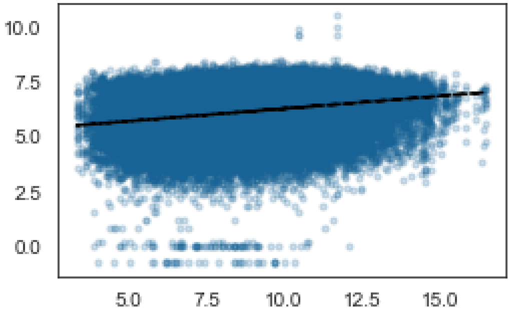

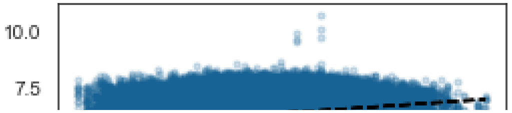

Create PDF in your applications with the Pdfcrowd HTML to PDF API PDFCROWD

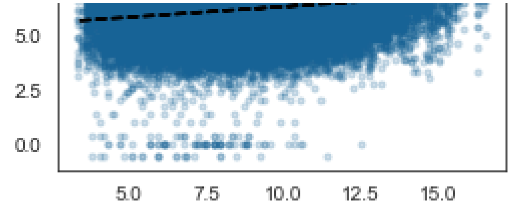

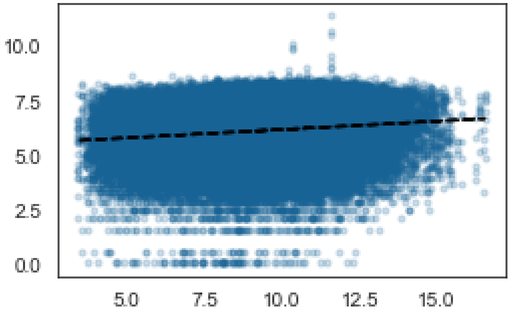

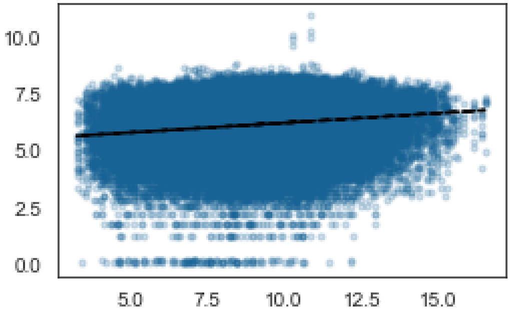

In [ ]:

```
x = by_gene_rna_broad[coln1[i]]+
y =
cor_log(x,y, 'Broad_10samples')
```

Create PDF in your applications with the Pdfcrowd HTML to PDF API PDFCROWD

In [ ]:
```
#BROAD CONCATENATED
x=[]
y=[]
for i in range(0, len(coln1)):
    x+=list(by_gene_rna_broad[coln1[i]].dropna().replace([np.inf, -np.i
nf], 0))
    y+=list(by_gene_rna_broad[coln2[i]].dropna().replace([np.inf, -np.i
nf], 0))
#x=np.array([[value,float(1)]for value in x])
#x=x.astype(np.float64)
#y=np.array([[value,float(1)]for value in y])
#y=y.astype(np.float64)
#df = pd.DataFrame(x,y)
#df
#print(scipy.stats.pearsonr(x,y))
cor_log(x,y, 'Broad_10samples')
```

In [ ]:

In [ ]:
```
#NUCL CONCATENATED
x=[]
y=[]
for i in range(0, len(coln1)):
    x+=list(by_gene_rna_nucl[coln1[i]].dropna().replace([np.inf, -np.in
f], 0))
    y+=list(by_gene_rna_nucl[coln2[i]].dropna().replace([np.inf, -np.in
f], 0))

cor_log(x,y, 'Nucleosomal_10samples')
```

In [ ]:

In [101]:
```
import seaborn as sns
sns.set_style("white")

# Import data
x=pd.Series(broad_r)
plt.figure(figsize = [4, 2.5])
```

Create PDF in your applications with the Pdfcrowd HTML to PDF API PDFCROWD

```
# Plot
kwargs = dict(hist_kws={'alpha':.6}, kde_kws={'linewidth':2})
# Plotting hist without kde
ax = sns.distplot(x, color="dodgerblue",bins = 5, kde=False, **kwargs)

# Creating another Y axis
second_ax = ax.twinx()

#Plotting kde without hist on the second Y axis
sns.distplot(x, ax=second_ax, color="dodgerblue", kde=True, hist=False)

#Removing Y ticks from the second axis
second_ax.set_yticks([])


#plt.legend();
plt.savefig('broad_rcor.png', transparent=True, bbox_inches="tight", dp
i = 500)
```

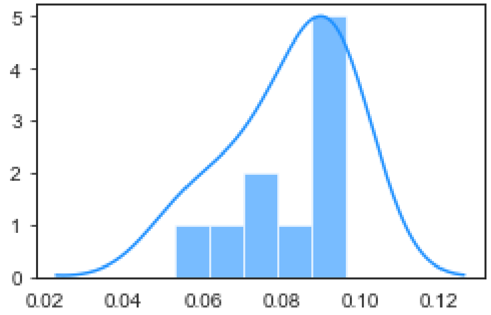

In [26]:
```
sns.distplot(broad_r, bins=5, norm_hist=True)
```

Out[26]: <matplotlib.axes._subplots.AxesSubplot at 0x22da8ed1b70>

Create PDF in your applications with the Pdfcrowd HTML to PDF API PDFCROWD

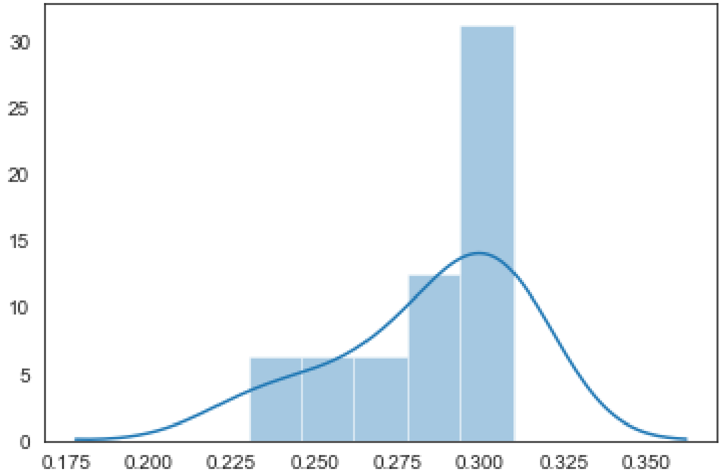

In [27]:

```
# Plotting hist without kde
ax = sns.distplot(x, kde=False)

# Creating another Y axis
second_ax = ax.twinx()

#Plotting kde without hist on the second Y axis
sns.distplot(x, ax=second_ax, kde=True, hist=False)

#Removing Y ticks from the second axis
second_ax.set_yticks([])
```

Out[27]: []

Create PDF in your applications with the Pdfcrowd HTML to PDF API PDFCROWD

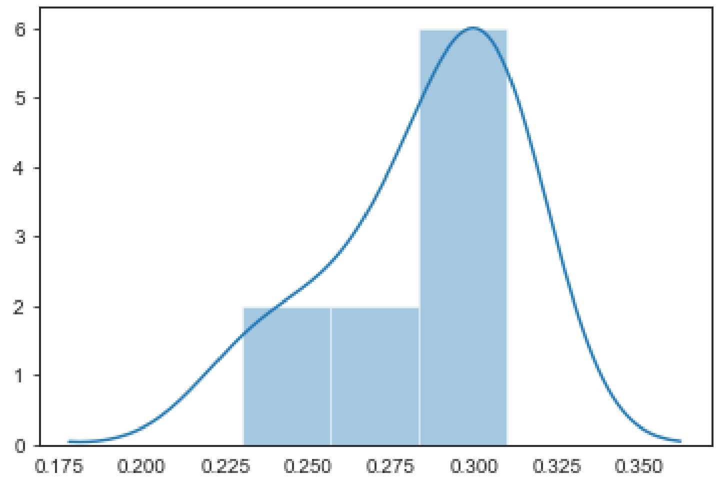

In [102]:

```
import seaborn as sns
sns.set_style("white")

# Import data
x=pd.Series(nucl_r)
plt.figure(figsize = [4, 2.5])
# Plot
kwargs = dict(hist_kws={'alpha':.6}, kde_kws={'linewidth':2})
# Plotting hist without kde
ax = sns.distplot(x, color="dodgerblue",bins = 5, kde=False, **kwargs)

# Creating another Y axis
second_ax = ax.twinx()

#Plotting kde without hist on the second Y axis
sns.distplot(x, ax=second_ax, color="dodgerblue", kde=True, hist=False)

#Removing Y ticks from the second axis
second_ax.set_yticks([])
#plt.legend();
plt.savefig('nucl_rcor.png', transparent=True, bbox_inches="tight", dpi
 = 500)
```

Create PDF in your applications with the Pdfcrowd HTML to PDF API PDFCROWD

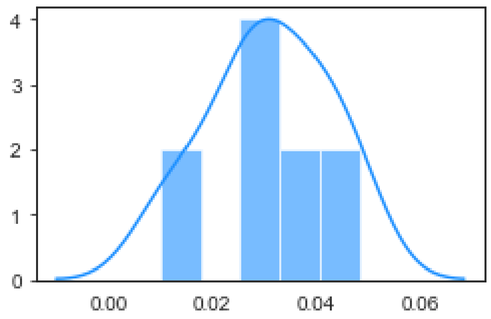

In [66]:
```
import matplotlib.mlab as mlab
x=by_gene_rna_broad['Pearson_cor_R']
# the histogram of the data
n, bins, patches = plt.hist(x, 5, facecolor='green', alpha=0.75)


plt.xlabel('Pearson, R')


plt.show()
```

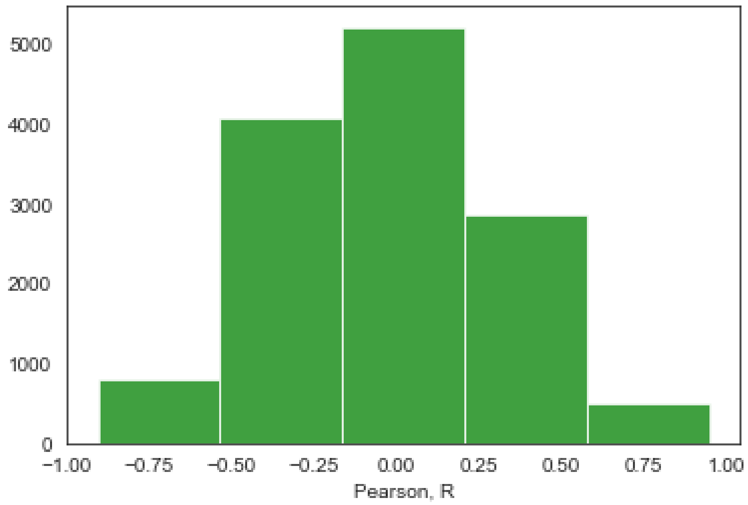

Create PDF in your applications with the Pdfcrowd HTML to PDF API PDFCROWD

In [68]:

```
# example data
plt.figure(figsize=(10,6))
x=by_gene_rna_broad['Pearson_cor_R']
mu = x.mean() # mean of distribution
sigma = x.std() # standard deviation of distribution
num_bins = 5
# the histogram of the data
n, bins, patches = plt.hist(x, num_bins, density=True, facecolor='blue'
, alpha=0.5)

# add a 'best fit' line
y = mlab.normpdf(bins, mu, sigma)
plt.plot(bins, y, 'r--')
plt.xlabel('Pearson, R')

# Tweak spacing to prevent clipping of ylabel
plt.subplots_adjust(left=0.15)

plt.yticks([])
plt.savefig('broad_cor_by_gene.png', transparent=True, bbox_inches="tig
ht")
```

```
C:\ProgramData\Anaconda3\lib\site-packages\ipykernel_launcher.py:11: Ma
tplotlibDeprecationWarning: scipy.stats.norm.pdf
  # This is added back by InteractiveShellApp.init_path()
```

Create PDF in your applications with the Pdfcrowd HTML to PDF API PDFCROWD

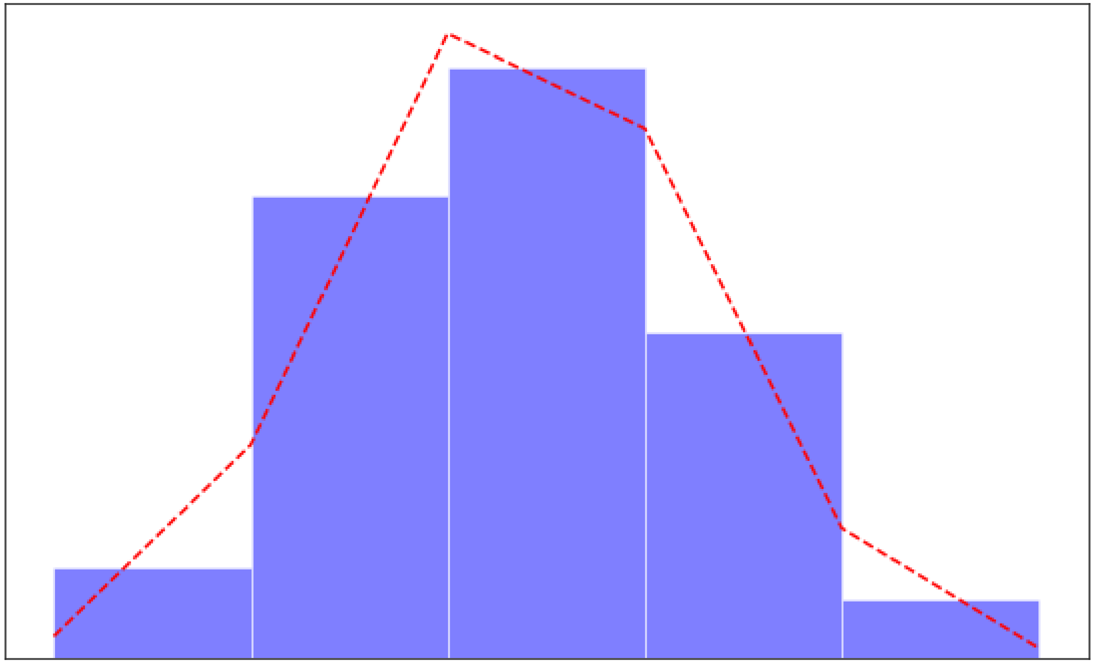

In [67]:

```
# example data
plt.figure(figsize=(10,6))
x=by_gene_rna_nucl['Pearson_cor_R']
mu = x.mean() # mean of distribution
sigma = x.std() # standard deviation of distribution
num_bins = 5
# the histogram of the data
n, bins, patches = plt.hist(x, num_bins, density=True, facecolor='blue'
, alpha=0.5)

# add a 'best fit' line
y = mlab.normpdf(bins, mu, sigma)
plt.plot(bins, y, 'r--')
plt.xlabel('Pearson, R')
```

Create PDF in your applications with the Pdfcrowd HTML to PDF API PDFCROWD

```python
# Tweak spacing to prevent clipping of ylabel
plt.subplots_adjust(left=0.15)

plt.yticks([])
plt.savefig('nucl_cor_by_gene.png', transparent=True, bbox_inches="tigh
t")
```

```
C:\ProgramData\Anaconda3\lib\site-packages\ipykernel_launcher.py:11: Ma
tplotlibDeprecationWarning: scipy.stats.norm.pdf
  # This is added back by InteractiveShellApp.init_path()
```

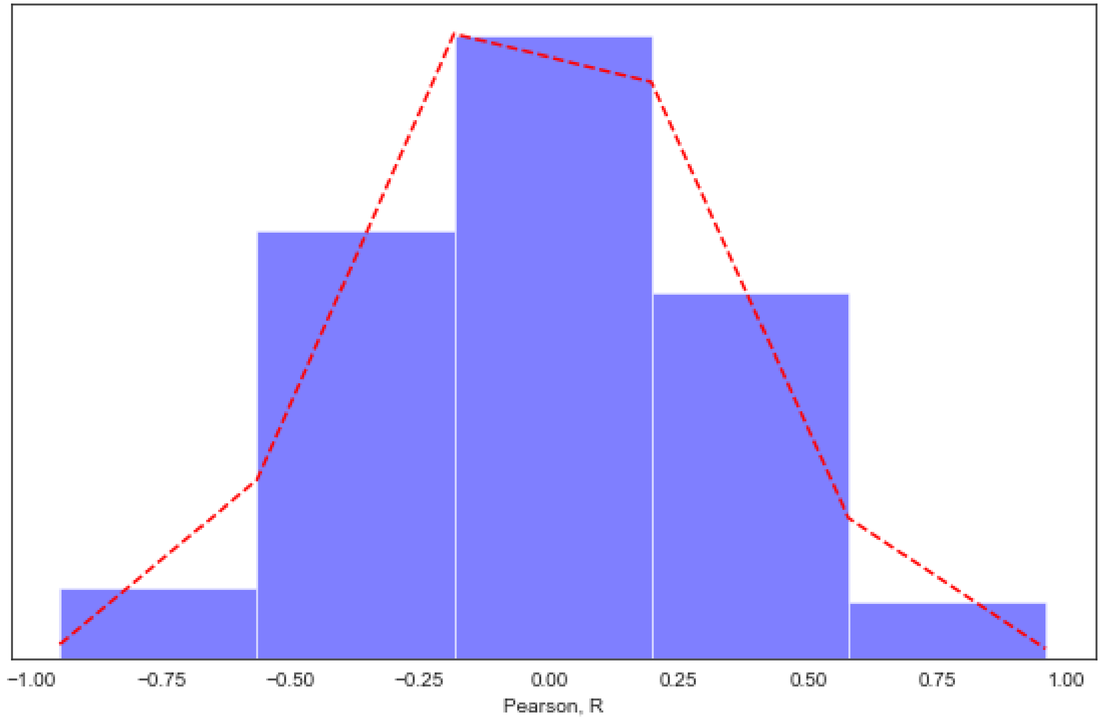

In [83]:
```python
by_gene_rna_broad
```

Out[83]:

	gene_name	K_RNA_204	K_RNA_205	K_RNA_208	K_SS_RNA_11	K_SS_RNA_15	K_SS_
0	Izumo4	280.443370	325.570300	302.694497	454.546689	514.327388	44

Create PDF in your applications with the Pdfcrowd HTML to PDF API PDFCROWD

	gene_name	K_RNA_204	K_RNA_205	K_RNA_208	K_SS_RNA_11	K_SS_RNA_15	K_SS_
1	Stim2	2112.854903	2240.570757	2246.063835	1696.562018	1802.431756	173
2	Col16a1	186.962247	153.685359	166.069743	372.089240	328.026578	37
3	Col11a2	219.635261	183.006908	201.403731	331.891233	350.885573	29
4	Echdc2	242.324854	197.162138	221.426325	330.860515	310.882332	36
...	...	...	...	...	...	...	
13462	Gm26673	74.421865	80.887031	38.867387	75.242422	49.146839	4
13463	Eepd1	467.405616	421.623649	394.562866	449.393098	389.745865	38
13464	Fam167a	24.504760	30.332637	18.844794	38.136570	20.573096	2
13465	Cdk16	6035.431746	4944.219767	5078.671870	5543.202023	5114.700132	490
13466	Zranb1	1718.963568	1926.122424	1990.481322	1723.360689	2093.883942	188

13467 rows × 26 columns

In [93]:
```
f = by_gene_rna_broad
print('gene number cor '+str(f.loc[f['Pearson_cor_pval']<0.05].count()[
0]))
print('% cor '+str(100*(f.loc[f['Pearson_cor_pval']<0.05].count()[0])/f
.count()[0]))
print('gene number cor <0 '+str(f.loc[(f['Pearson_cor_pval']<0.05) & (f
['Pearson_cor_R']<0)].count()[0]))
print('gene number cor >0 '+str(f.loc[(f['Pearson_cor_pval']<0.05) & (f
['Pearson_cor_R']>0)].count()[0]))
```

```
gene number cor 688
% cor 5.108784436028811
gene number cor <0 372
gene number cor >0 316
```

In [94]:
```
f = by_gene_rna_nucl
print('gene number cor '+str(f.loc[f['Pearson_cor_pval']<0.05].count()[
0]))
```

Create PDF in your applications with the Pdfcrowd HTML to PDF API PDFCROWD

```
print('% cor '+str(100*(f.loc[f['Pearson_cor_pval']<0.05].count()[0])/f
.count()[0]))
print('gene number cor <0 '+str(f.loc[(f['Pearson_cor_pval']<0.05) & (f
['Pearson_cor_R']<0)].count()[0]))
print('gene number cor >0 '+str(f.loc[(f['Pearson_cor_pval']<0.05) & (f
['Pearson_cor_R']>0)].count()[0]))
```

```
gene number cor 3528
% cor 4.803005962915567
gene number cor <0 1904
gene number cor >0 1624
```

In [23]:

```
by_gene_rna_nucl.to_excel('nucleosomal-peaks_rnaseq_CSDS_corelation.xls
x')
by_gene_rna_broad.to_excel('broad-peaks_rnaseq_CSDS_corelation.xlsx')
```

In [96]:

```
by_gene_rna_nucl.loc[by_gene_rna_nucl['Pearson_cor_pval']<0.05].to_exce
l('nucleosomal-peaks_rnaseq_CSDS_corelation005.xlsx')
by_gene_rna_broad.loc[by_gene_rna_broad['Pearson_cor_pval']<0.05].to_ex
cel('broad-peaks_rnaseq_CSDS_corelation005.xlsx')
```

In [98]:

```
spec = pd.read_excel('CELL_SPECIFIC SCIENTIFIC REPORTS 2018.xlsx', shee
t_name = 'top_mouse_specificity')
spec_expr = pd.read_excel('CELL_SPECIFIC SCIENTIFIC REPORTS 2018.xlsx',
 sheet_name = 'top_mouse_expression')
```

In [132]:

```
spec100 = pd.read_excel('CELL_SPECIFIC SCIENTIFIC REPORTS 2018.xlsx', s
heet_name = 'spec100')
```

In [99]:

```
ge = [i.upper() for i in list(by_gene_rna_broad['gene_name'])]
by_gene_rna_broad['gene_name_upper'] = ge
by_gene_rna_broad
```

Out[99]:

	gene_name	K_RNA_204	K_RNA_205	K_RNA_208	K_SS_RNA_11	K_SS_RNA_15	K_SS_
0	Izumo4	280.443370	325.570300	302.694497	454.546689	514.327388	44

Create PDF in your applications with the Pdfcrowd HTML to PDF API PDFCROWD

	gene_name	K_RNA_204	K_RNA_205	K_RNA_208	K_SS_RNA_11	K_SS_RNA_15	K_SS_
1	Stim2	2112.854903	2240.570757	2246.063835	1696.562018	1802.431756	173
2	Col16a1	186.962247	153.685359	166.069743	372.089240	328.026578	37
3	Col11a2	219.635261	183.006908	201.403731	331.891233	350.885573	29
4	Echdc2	242.324854	197.162138	221.426325	330.860515	310.882332	36
...	...	...	...	...	...	...	
13462	Gm26673	74.421865	80.887031	38.867387	75.242422	49.146839	4
13463	Eepd1	467.405616	421.623649	394.562866	449.393098	389.745865	38
13464	Fam167a	24.504760	30.332637	18.844794	38.136570	20.573096	2
13465	Cdk16	6035.431746	4944.219767	5078.671870	5543.202023	5114.700132	490
13466	Zranb1	1718.963568	1926.122424	1990.481322	1723.360689	2093.883942	188

13467 rows × 27 columns

In [133]:
```python
ch1 = pd.merge(by_gene_rna_broad, spec100[['gene', 'Celltype']], left_on = 'gene_name_upper', right_on = 'gene')
ch1
```

Out[133]:

	gene_name	K_RNA_204	K_RNA_205	K_RNA_208	K_SS_RNA_11	K_SS_RNA_15	K_SS_RN
0	Gfap	1728.039405	1491.354633	2015.215113	2495.368557	2255.039857	2676.53
1	Slc27a1	1166.245082	1012.098975	1003.485258	1414.145254	1187.524790	1314.33
2	Itga7	115.263133	88.975734	98.935166	148.423409	118.866774	147.77
3	Fzd2	235.971768	194.128874	248.515715	316.430461	272.022041	251.83
4	Fgfr3	1516.572398	1373.057350	1492.272092	1923.320003	1606.987349	1552.63
...	...	...	...	...	...	...	
62	Fjx1	1603.700435	1234.538310	1435.737711	1584.213743	1408.114092	1304.96
63	Bmpr1b	469.220784	580.364447	699.612962	620.492305	574.903724	570.27

Create PDF in your applications with the Pdfcrowd HTML to PDF API PDFCROWD

	gene_name	K_RNA_204	K_RNA_205	K_RNA_208	K_SS_RNA_11	K_SS_RNA_15	K_SS_RN
64	Chrdl1	687.948461	829.092067	1134.221014	911.154814	926.932247	765.91
65	Ezr	1336.870821	1405.412163	1434.559911	1444.036079	1429.830137	1202.98
66	P4ha3	68.068779	86.953558	69.490176	89.672476	78.863533	57.23

67 rows × 29 columns

In [112]:

```
tab1 = pd.DataFrame()
```

In [135]:

```
def cpec_graph(df, name):
    sample = []
    cell = []
    pearcor = []
    pval = []
    gn=[]
    pn=[]
    for i in set(df['Celltype']):
        plt.figure()
        sp=df.loc[ch1['Celltype']==i]
        x= np.log2(sp[sp.columns[0]]).replace([np.inf, -np.inf], 0)
        y = np.log2(sp[sp.columns[1]]).replace([np.inf, -np.inf], 0)
        pc = scipy.stats.pearsonr(x,y)
        print(i+'\n'+str(pc))

        pn.append(len(sp['gene_name']))
        gn.append(len(set(sp['gene_name'])))
        pearcor.append(pc[0])
        pval.append(pc[1])
        sample.append([sp.columns[0]])
        cell.append(i)

        print(len(set(sp['gene_name'])))
        plt.scatter(x, y, c = 'black', alpha = 0.4, s = 14)
        # calc the trendline
        z = np.polyfit(x, y, 1)
```

Create PDF in your applications with the Pdfcrowd HTML to PDF API PDFCROWD

```
        p = np.poly1d(z)
        plt.plot(x,p(x),"r--")
        plt.title(i)
        # the line equation:
        print("y=%.6fx+(%.6f)"%(z[0],z[1]))
        plt.savefig(f'{i}{name}.png', transparent=True, bbox_inches="ti
ght")
    tab = {'mice':sample,'celltype':cell, 'Pearson coefficient': pearco
r, 'P-val':pval, 'peak number':pn, 'gene number':gn}
    pd.DataFrame(tab).to_excel(f'{name}.xlsx')
```

In [136]:

```
for i in range(0, len(coln1)):
    cpec_graph(ch1[[coln1[i], coln2[i], 'Celltype', 'gene_name']], 'Bro
ad_'+str(coln1[i]))
```

```
ast
(0.1896256027683212, 0.12431195466613147)
66
y=0.141113x+(6.801468)
ast
(0.24657306531598275, 0.04427489025920177)
66
y=0.180890x+(6.476630)
ast
(0.12190423687889092, 0.32574410554790234)
66
y=0.096617x+(7.179109)
ast
(0.22385717229804344, 0.06859879531606496)
66
y=0.160924x+(6.626286)
ast
(0.17109367584686871, 0.1662548805006512)
66
y=0.135723x+(6.797269)
ast
(0.14751247240346446, 0.2335559367032898)
66
y=0.122391x+(6.930746)
```

Create PDF in your applications with the Pdfcrowd HTML to PDF API PDFCROWD

```
ast
(0.21191579886959985, 0.08514664683747139)
66
y=0.162467x+(6.519756)
ast
(0.24171978598455282, 0.0487631950696183)
66
y=0.174707x+(6.483758)
ast
(0.14385461118124956, 0.2454817302559003)
66
y=0.119966x+(6.868787)
ast
(0.1980679303656756, 0.10811847568530414)
66
y=0.156329x+(6.670025)
```

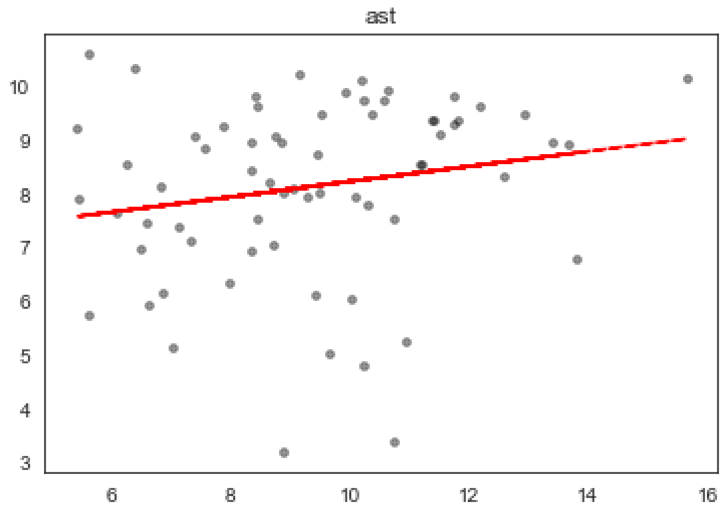

Create PDF in your applications with the Pdfcrowd HTML to PDF API PDFCROWD

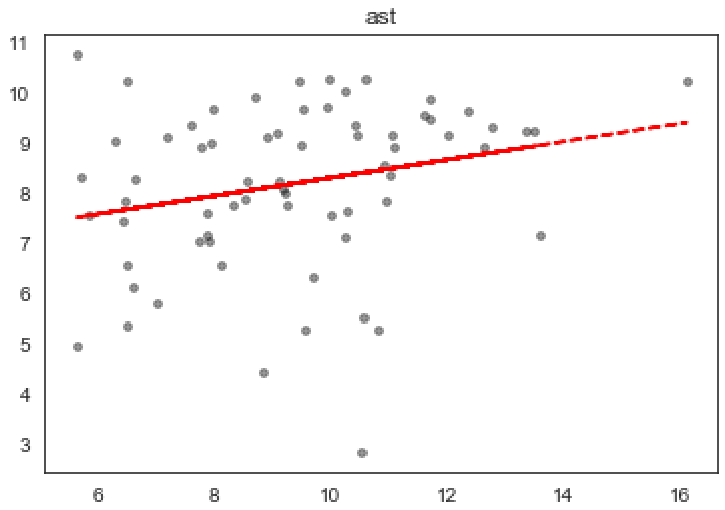

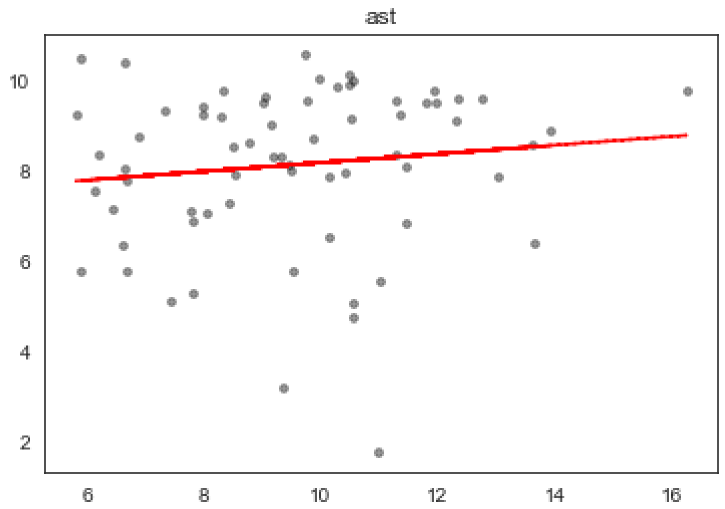

Create PDF in your applications with the Pdfcrowd HTML to PDF API
PDFCROWD

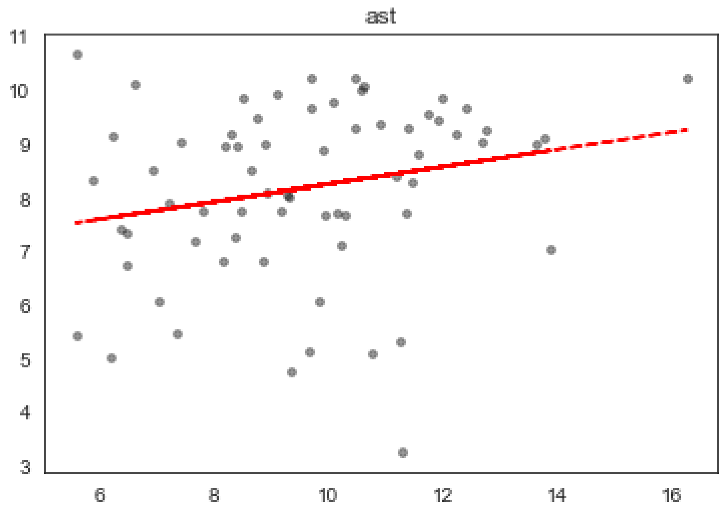

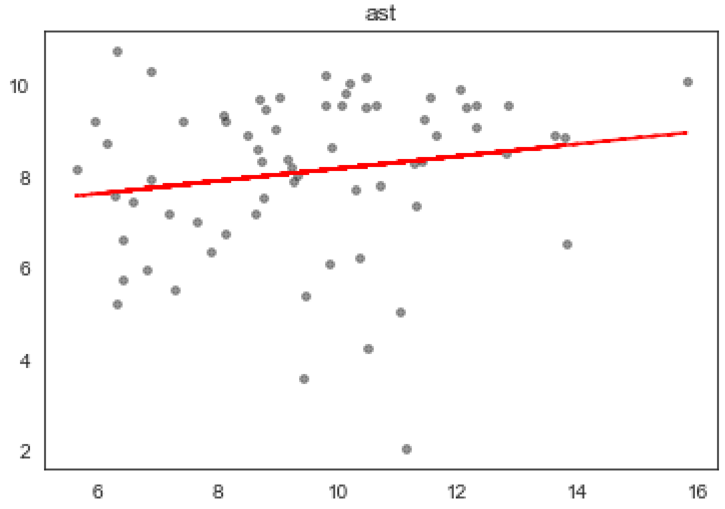

ast

Create PDF in your applications with the Pdfcrowd HTML to PDF API PDFCROWD

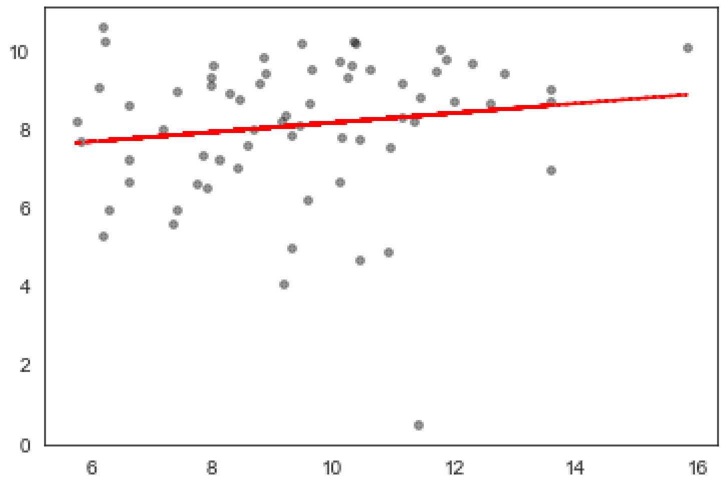

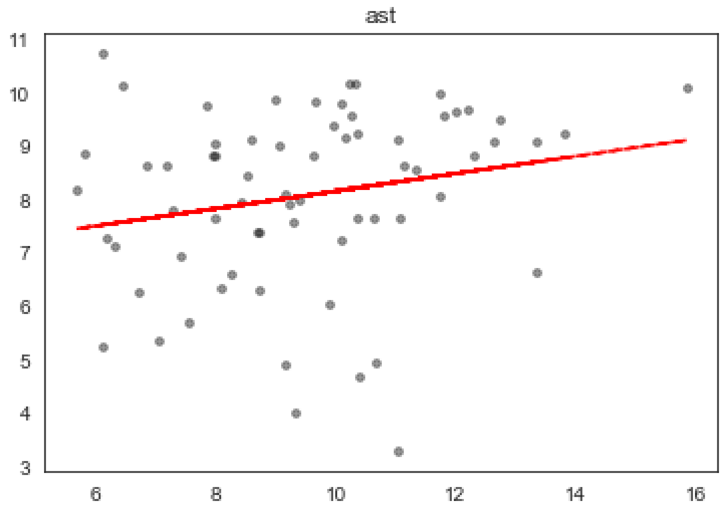

Create PDF in your applications with the Pdfcrowd HTML to PDF API PDFCROWD

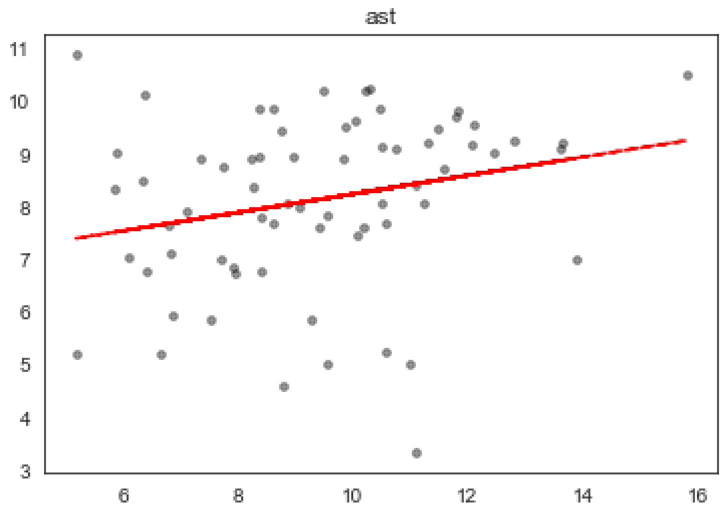

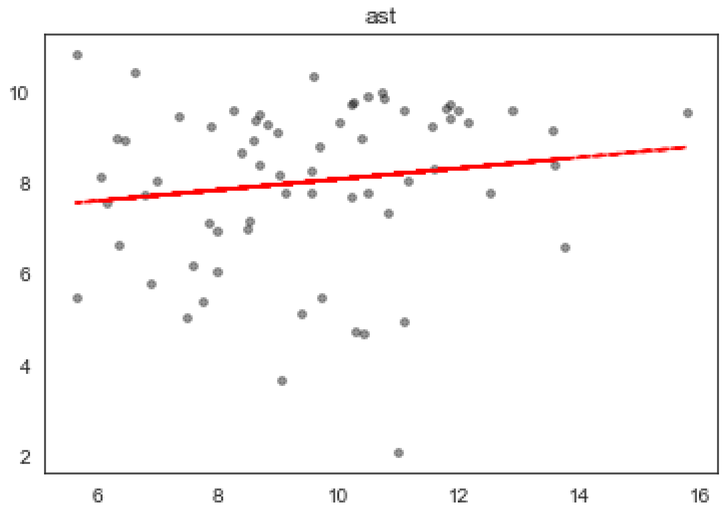

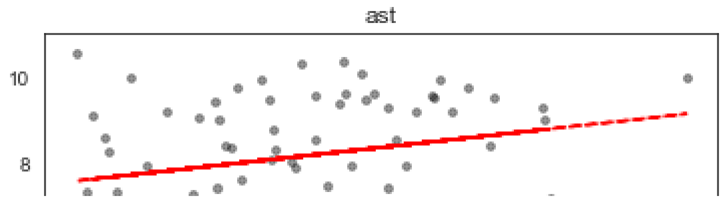

Create PDF in your applications with the Pdfcrowd HTML to PDF API PDFCROWD

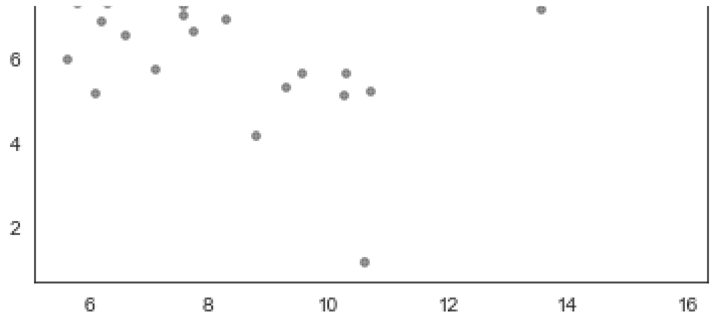

Create PDF in your applications with the Pdfcrowd HTML to PDF API
PDFCROWD
